# Supplementary material for: Identification of photoperiod-induced specific miRNAs in the adrenal glands of Sunite sheep (Ovis aries)
Source: Front Vet Sci. 2022 Jul 22;9:888207. doi: 10.3389/fvets.2022.888207 (PMC9354845; doi:10.3389/fvets.2022.888207)
Supplement: Supplementary file 1 [file Data_Sheet_1.ZIP › Supplementary file/miRDeep2 file.zip!/miRDeep2 file/Know_Structure/SPLP42A2/oar-mir-106b.pdf]

miRBase precursor : oar-mir-106b
Total read count : 4551
oar-miR-106b read count : 1567
remaining reads : 2984

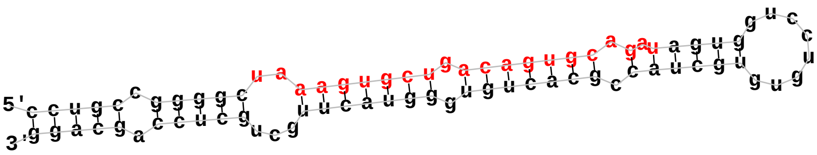

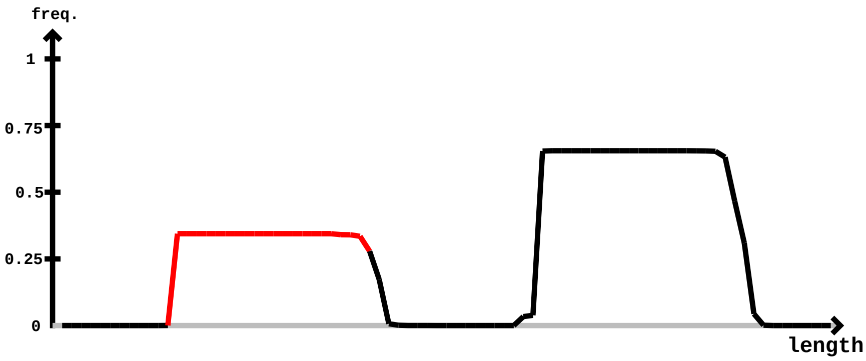


```
                 oar-miR-106b
5'- ccugccggggcuaaagugcugacagugcagauagugguccugugugcuaccgcacuguggguacuugcugcuccagcagg   -3'    exp
    (((((.(((((..(((((((.(((((((.(.(((((........)))))).))))))).)))))))...))))).)))))    reads      mm         sample
    ..........cuaaagugcugacagugcaga.................................................    2          0          seq
    ...........uaaaAugcugacagugc....................................................    1          1          seq
    ...........uaaagugcugacagugc....................................................    15         0          seq
    ...........uaaagugcugacagugA....................................................    1          1          seq
    ...........uaaagugcugacagugca...................................................    2          0          seq
    ...........uaaagugcugacagugcag..................................................    25         0          seq
    ...........uaaagugcugacagugcaga.................................................    252        0          seq
    ...........uaaagugcugacagugcagau................................................    484        0          seq
    ...........uaaagugcugacagugcagaua...............................................    755        0          seq
    ...........uaaagugcugacagugcagauag..............................................    25         0          seq
    ...........uaaagugcugacagugcagauagu.............................................    5          0          seq
    ...........uaaagugcugacagugcagauaguggu...........................................    1          0          seq
    ...............................................uaccgcacuguggguacu...............    1          0          seq
    ...............................................uaccgcacuguggguacuu..............    1          0          seq
    ...............................................uaccgcacuguggguacuug.............    1          0          seq
    ...............................................uaccgcacuguggguacuugc............    11         0          seq
    ...............................................uaccgcacuguggguacuugcu...........    137        0          seq
    ...............................................uaccgcacuguggguacuugcug..........    1          0          seq
    ...............................................uaccgcacuguggguacuugcugc.........    1          0          seq
    ................................................accgcacuguggguacuugcu...........    13         0          seq
    ................................................accgcacuguggguacuugcug..........    6          0          seq
    ................................................accgcacuguggguacuugcugc.........    3          0          seq
    .................................................ccgcacuguggguacuu..............    1          0          seq
    .................................................ccgcacuguggguacuug.............    5          0          seq
    .................................................ccgcacuguggguacuugc............    89         0          seq
    .................................................ccgcacuguggguacuugcu...........    600        0          seq
    .................................................ccgcacuguggguacuugcug..........    707        0          seq
    .................................................ccgcacuguggguacuugcugc.........    1202       0          seq
    .................................................ccgcacuguggguacuugcugcu........    193        0          seq
    .................................................ccgcacuguggguacuugcugcuc.......    6          0          seq
    ..................................................cgcacuguggguacuugcu...........    1          0          seq
    ..................................................cgcacuguggguacuugcug..........    1          0          seq
    ..................................................cgcacuguggguacuugcugc.........    2          0          seq
    ..................................................cgcacuguggguacuugcugcu........    1          0          seq
```
